# Finite analysis of stability between modified articular fusion technique, posterior lumbar interbody fusion and posteriorlateral lumbar fusion

**DOI:** 10.1186/s12891-021-04899-x

**Published:** 2021-12-04

**Authors:** Xiao Han, Xin Chen, Kuan Li, Zheng Li, Shugang Li

**Affiliations:** Department of Orthopaedics, Peking Union Medical College Hospital, Chinese Academy of Medical Sciences and Peking Union Medical College, Dongcheng District Shuaifuyuan No. 1, Beijing, 100730 China

**Keywords:** Finite element, Spinal fusion, Internal fixation

## Abstract

**Background:**

It is not clear whether modified facet fusion (MFF) is biomechanically different from traditional fusion techniques such as posterior lateral lumbar fusion (PLF) and posterior lumbar interbody fusion (PLIF).

**Methods:**

In this study, a healthy adult Chinese male volunteer was selected to perform 3D reconstruction of CT image data and simulate the successful fusion of L4–5 MFF, PLF and PLIF, respectively. The motion range of L4–5 segments of the model was simulated under 6 working conditions, including forward flexion, extension, lateral flexion and rotation under normal physiological conditions, and the stability of the three fusion procedures in the pathological segments of the lumbar spine was compared.

**Results:**

There was no difference in range of motion between MFF model and PLF or PLIF model (*P* < 0.05). Also, the stiffness of the PLFand the MFF model were comparable (*P* > 0.05), but were smaller than the PLIF model (*P* < 0.05).

**Conclusions:**

MFF provides reliable stability at the lumbar fixation fusion level and does not differ significantly from PLF and PLIF in terms of range of motion.

## Introduction

The concept of “finite element” has been proposed for more than 70 years [1]. Early used in structural design and analysis of some continuum mechanics and physical problems, it is an important method in mechanical calculation [2]. In 1973, Belytschko et al. [3] first applied the finite element analysis method to the study of spine biomechanics, marking the beginning of the application of finite element method in orthopedic biomechanical analysis. Hakim and King [4] then added a posterior attachment structure to the single lumbar spine model to analyze the biomechanical characteristics under static and dynamic conditions. The finite element method expresses the structural shape, material properties and load conditions of the spine mathematically and reveals the influence on the entire structure by variance of any of the parameters [5, 6]. More and more scholars have begun to apply finite element analysis to investigate mechanical changes in the physiological and pathological processes of the spine, as well as the working principle and stress distribution characteristics of various internal fixation devices in order to provide a basis for clinical improvement and optimization of the surgical plan of the spine [7–9].

In the surgical treatment of lumbar degenerative diseases, spinal decompression is the most commonly used treatment technique in clinical practice [10]. In order to obtain a satisfactory surgical effect, adequate decompression of the spinal canal will often partially or completely remove the bilateral intervertebral joints, resulting in loss of stability of the segment. In order to prevent complications such as lumbar instability caused by surgery, biomechanical reconstruction is often required. Therefore, it is often necessary to use internal fixation instruments and bone graft fusion techniques to reconstruct and maintain lumbar stability [11]. However, no matter what kind of internal fixation device is used, only short-term stability can be obtained. In order to make patients obtain reliable and stable long-term, successful bone graft fusion plays a decisive role [12].

Our published preliminary study has shown that modified articular bone grafting under internal fixation is a fusion technique with high fusion rate and advantage of relatively simple to operate [13], and it can achieve similar surgical results as posterior lumbar interbody fusion (PLIF) does in previous studies [14, 15]. However, the MFF only fuses the posterior column structure of the spine, while the PLIF fuses the main load structure such as the anterior middle column of the spine. Currently there is no relevant research compare the difference in biomechanics between the MFF, PLF and PLIF.

In this study, a three-dimensional finite element model of human lumbar vertebrae was established by finite element method. Consequently, successful lumbar spine model of MFF, PLF and PLIF was established. The comparison of biomechanical data among these models would help analyze the process of motion and provide surgeons with theoretical evidence to select suitable surgical technique.

## Materials and methods

1. Study Object

One healthy adult Chinese male volunteer (30 years old, 178 cm in height, 75 kg in weight, without history of spinal disease or spinal trauma) took part in the study after signing an informed consent. He further took an X-ray examination to confirm the presence or absence of spinal deformity and other spinal diseases.

2. Model establishment of PLF spinal decompression

**Fig. 1:**
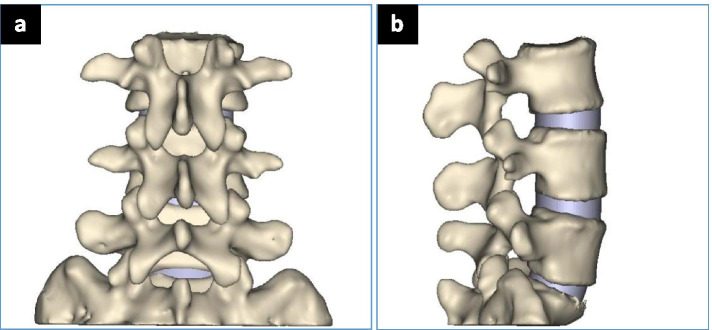
Intact Lumbar Model. **a** AP view; **b** LAT view

Three-dimensional CT image reconstruction of vertebrae from L1 to S1 was performed using the interactive medical image control system MIMICS 17.0 software. The lumbar vertebrae of the subject were scanned with a 64-row spiral CT by General Engine and a total of 346 layers of images were obtained (voltage 120 kV, current 200 mA, scanning layer thickness 0.625 mm, layer spacing 0.625 mm)in DICOM format. Then the image was imported into the MIMICS software, and the two-dimensional data of the L3 ~ S1 segment was reconstructed into three-dimensional data. In this study, each vertebral body and intervertebral disc was used as independent entities and they were cross-referenced in a three-dimensional window. The human three-dimensional images of the vertebral bodies and intervertebral discs of the L3 to S1 were reconstructed by manual cutting. Intact Lumbar Model is shown in Fig. 1.

On the basis of normal three-dimensional images, the MIMICS software was used to simulate the posterior surgical procedure according to the usual resection range of posterior lumbar spinal decompression surgery in clinical practice and the corresponding structure of the degenerative segment (L4–5) was removed to establish a postoperative model from L4 to L5:

1. PLF and MFF models: the resection range was 2/3 of the inferior L4 lamina, medial 2/3 of the L4 bilateral inferior articular process, 1/5 of upper L5 lamina and 1/3 of the medial side of bilateral superior articular processes of L5, as well as the corresponding supraspinous ligament, interspinous ligament, ligamentum flavum and joint capsule;
2. PLIF model: the resection range was entire lamina of L4, medial side of L4 bilateral inferior articular processes, upper 1/5 of L5 lamina, and medial part of L5 bilateral superior articular processes, as well as the corresponding supraspinous ligament and interspinous ligaments, ligamentum flavum and joint capsules.

The above three-dimensional image was then imported into the reverse engineering software Geomagic 2013 for optimization. Firstly, the noise point in the point cloud image was reduced under the premise of ensuring the integrity of the model. Then the encapsulation was completed by using the point cloud image, and the triangular patch was reduced under the premise of ensuring the shape of the model. Next, the voids were filled which should not exist on the surface of the model, remove non-characterized spikes and dents on the surface of the model, smooth and relax the surface, prevent the occurrence of non-characteristic high curvature and self-intersecting surfaces, so as to avoid subsequent meshing Unnecessary low-quality meshes, which implement a smooth NURBS surface to fit discrete model surface triangle patches, and finally generate a solid model.

### 3D model of surface mesh was transformed into 3D model of volume mesh

The solid model built in the Geomagic software was imported into the ABAQUS software in igs format, and the mesh is generated by the Mesh tool in the ABAQUS preprocessor and further converted into a volume mesh.

### Defining materials and section properties

Based on previous studies, the material and section properties for the parameters of the cortical bone, cancellous bone, cartilage endplate, pedicle, transverse process, spinous process, lamina, and intervertebral disc annulus and nucleus of the vertebral body were defined (Table 1). The degenerative segment (L4–5) intervertebral disc (annulus and nucleus) was defined as the degenerative material property, while the L3–4 and L5-S1 intervertebral discs were defined as normal material properties [16, 17]. The main ligaments such as anterior longitudinal ligament, posterior longitudinal ligament, supraspinous ligament, interspinous ligament, ligamentum flavum, joint capsule, and intertransverse ligament were established. In this study, the ligament structure was simulated by spring unit and referenced to previous literature to define the material properties of the above ligaments [18, 19] (Table 2).

**Table 1:** Lumbar finite element model: unit type and material properties

Structure	Unit Type	Thickness(mm)	Elastic Modulus(MPa)	Poisson’s ratio
Vertebra cortical bone	S3R	0.4	12,000	0.3
Vertebra cancellous bone	C3D4	N/A	100	0.2
Vertebra endplate cartilage	S3R	0.25	1000	0.2
Vertebra posterior structure	C3D4	N/A	3500	0.25
Fibrous annulus(Normal)	C3D4	N/A	2.6	0.4
Nucleus pulposus(Normal)	C3D4	N/A	1.0	0.49
Fibrous annulus(Degenerative)	C3D4	N/A	12.3	0.35
Nucleus pulposus(Degenerative)	C3D4	N/A	1.7	0.4

**Table 2:** Lumbar finite element model main ligament material properties

Ligament	Elastic Modulus(MPa)	Cross-sectional area (mm^2^)	Average Length(mm)	Stiffness(kg•m^−2^•s^− 2^)
ALL	7.8	22.4	20	8.74
PLL	10	7.0	12	5.83
LF	17	14.1	15	15.83
TIL	10	0.6	32	0.19
JCL	7.5	10.5	5	15.75
IVL	10	14.1	13	10.85
SVL	8.0	10.5	22	2.39

*ALL* anterior longitudinal ligament, *PLL* posterior longitudinal ligament, *LF* ligamentum Flavum *TIL* Transverse intersegmental ligament, *JCL* Joint capsule ligament, *IVL* Intervertebra ligament, *SVL* Supravertebra ligament

### Establishment of geometric solid model for internal fixation system

According to pedicle diameter and distance from locating point to anterior border of vertebra, measured by CT, the internal fixation material required for the simulated operation was titanium polyaxial pedicle screw (6.5 mm in diameter, 50 mm long, Legacy, Medtronic, USA), and the interbody Cage is PEEK material (provided by Johnson & Johnson). Using the modeling function of the reverse engineering software Geomagic, the connecting rod and lumbar pedicle screw were drawn. The nail cap and the figure of the nail body are used to remove a small part of the curved surface and the thread without affecting the analysis of the mechanical properties of the next step, and the solid model of the connecting rod, the lumbar pedicle screw cap and the nail body was constructed according to the structure.

### Reconstruction of lumbar posterior internal fixation fusion model

We introduced the model of each component of the lumbar posterior internal fixation system into HyperMesh 14.0. Four internal fixation systems were inserted into the L4 and L5 pedicles with reference to the pedicle screw internal fixation technique. Next, visual adjustment to complete spatial position assembly in the same coordinate system was completed.

#### Lumbar pedicle screw technology: positioning, orientation, depth

1. Positioning: the intersection point between the horizontal line of the midpoint of the transverse process and the perpendicular line of the outer edge of the upper joint was the screw insertion point;
2. Orientation: The appropriate tip and tip angle of the screw was selected so that the screw is parallel to the end plate. The pedicle screw was positioned with a transverse angle of 10° to 15° to the sagittal plane;
3. Depth: The insertion depth of the screw was 80% of the depth of the vertebral body.

#### Establishment of three-dimensional model of lumbar posterior internal fixation

According to the fusion principle of the three fusion techniques, a three-dimensional model of posterior lumbar posterior internal fixation was established. The bone graft simulates the condition of bony fusion.

**Fig. 2:**
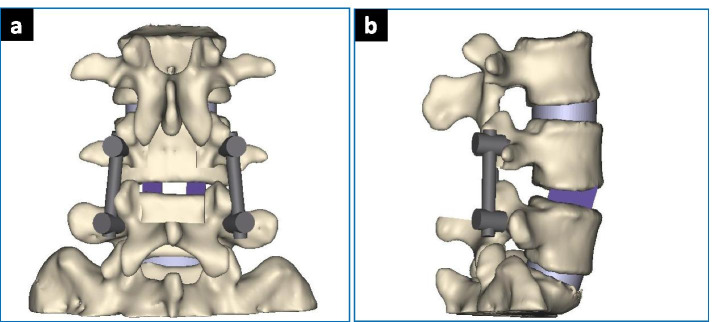
PLIF Model. **a** AP View; **b** LAT View

**Fig. 3:**
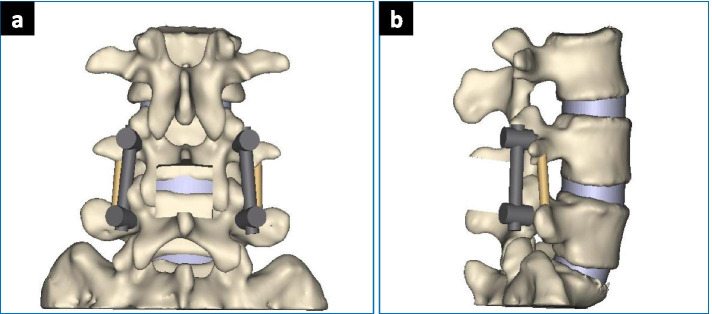
PLF model. **a** AP view; **b** LAT view

**Fig. 4:**
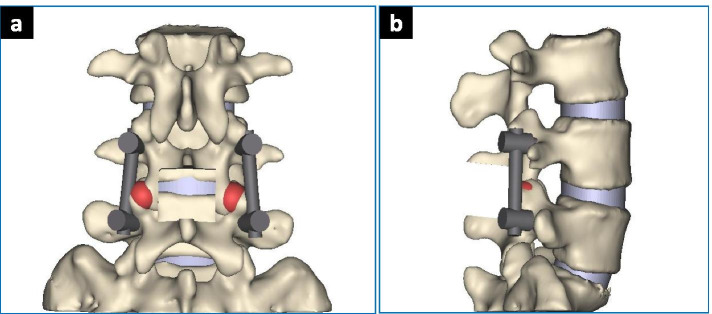
MFF model. **a** AP view; **b** LAT view

1. PLIF: L4/5 interbody fusion was performed after processing the upper and lower endplates of the L4–5 intervertebral space. The bone fusion was performed with cancellous bone (Fig. 2).
2. PLF: After processing the fusion interface of the bilateral transverse process and lateral cortical bone of the articular process, bone graft fusion with cancellous bone was performed to achieve L4/5 bilateral intertransverse fusion (Fig. 3).
3. MFF: First, we processed the inter-articular space as grafting bed as mentioned previously. Then bone fusion was conducted with cancellous bone (Fig. 4)

The models were again introduced into the reverse engineering software Geomagic for trimming and optimization, and therefore the solid modeling of the surgical conditions was completed. The data model was imported into the finite element analysis software ABAQUS. Boolean operation was performed between the bone and the screw, and the nail path after the screw was screwed into the bone is simulated on the model, and then the parts were meshed.

In the constructed three-dimensional finite element mesh model, the eigenvalues ​​are input into the model according to the elastic modulus and Poisson’s ratio of the implant material (Table 3). Then three-dimensional finite element model was reconstructed after posterior lumbar spinal decompression after successful fusion of different fusion techniques.

**Table 3:** Material properties of different parts of the internal fixation system

Internal Fixation System	Elastic Modulus(MPa)	Poisson’s ratio
Legacy Pedicle Screw	110,000	0.3
Legacy Connecting Rod	110,000	0.3
Johnson-johnson Cage(PEEK)	3600	0.3

### Setting boundary conditions and loads

During the analysis, the S1 vertebral body was fixed and the boundary conditions were set as the bottom of the S1 vertebral body and the bottom of the posterior structure were fixed, without displacement or rotation. A pressure of 500 N was applied to the endplate of the L3 vertebral body to simulate the load of the human body on the lumbar vertebrae in the static state of the human body. In addition, a pure moment of 10 Nm was applied to the endplate of the L3 vertebral body, which could cause the lumbar spine to move within the physiological range without destroying its organizational structure [16, 20]. According to the above constraints and loading conditions, the relevant data was imported into ABAQUS finite element software for analysis. Range of motion of the lumbar vertebrae under physiological conditions was simulated: flexion, extension, left and right lateral curvature, left and right rotation. The stiffness and stress distribution of each model under vertical physiological load conditions were also included.

#### Forward flexion and extension loading

On the basis of the axial 500 N load, plus the X-axis bending moment of 10 Nm, flexion-extension movement of the normal intact lumbar spine model, the posterior L4–5 spinal canal decompression + PLF model, the posterior L4–5 spinal canal decompression + PLIF, and posterior L4–5 spinal canal decompression + MFF model were simulated, respectively. As the moment was imposed on a random point within about 5 mm diameter, the above loading model was repeated 10 times to reduce statistical error, and the range of motion of the L4–5 segment after the corresponding load was measured and recorded.

#### Lateral flexion loading

On the basis of the axial 500 N load, plus the Z-axis bending moment of 10 Nm, lateral flexion movement of the normal intact lumbar spine model, the posterior L4–5 spinal canal decompression + PLF model, the posterior L4–5 spinal canal decompression + PLIF, and posterior L4–5 spinal canal decompression + MFF model were simulated, respectively. The above loading model was also repeated 10 times, and the range of motion of the L4–5 segment after the corresponding load was measured and recorded.

#### Rotating loading

On the basis of the axial 500 N load, plus the Y-axis bending moment of 10 Nm, axial rotational movement of the normal intact lumbar spine model, the posterior L4–5 spinal canal decompression + PLF model, the posterior L4–5 spinal canal decompression + PLIF, and posterior L4–5 spinal canal decompression + MFF model were simulated, respectively. Similarly, the above loading model was repeated 10 times, and the range of motion of the L4–5 segment after the corresponding load was measured and recorded.

#### Stiffness and stress distribution

On the basis of the axial 500 N physiological load, the posterior L4–5 spinal canal decompression + PLF inter-transverse fusion model, posterior L4–5 spinal canal decompression + PLIF model, posterior L4–5 spinal canal decompression + MFF mode were simulated to analyze stiffness and stress distribution. Then the above loading model was repeated 10 times.

#### Statistical methods

Shapiro–Wilk normality test was firstly performed to check the normality. Normally distributed data were tested with variance analysis of multiple independent samples and shown in the form of mean (standard deviation). Data that were not normally distributed was shown as median (range), and was tested by nonparametric Wilcoxon rank-sum test for multiple independent samples.

## Results

### Validation

A pure torque of 10 Nm was applied to the endplate of the L3 vertebral body on the L3 to S1 model, and the range of motion of the L4–5 segment flexion, extension, left and right lateral flexion, and left and right rotation was obtained: flexion 5.6 ± 1.3°, extension 4.6 ± 1.1°, side bend 3.4 ± 1.2°, rotation 2.3 ± 0.8°. This result was similar to the data measured by Panjabi et al. [21] on gross specimens, indicating that the model established in this study can better simulate the motion of real lumbar vertebrae.

### Comparison of the stability between the intact model and different fusion models

The range of activity of the L4–5 segment of the intact model and the three different fusion post-operative models under various loading conditions was shown in Table 4. The range of flexion, extension, lateral flexion, and rotation of the intact model L4–5 segment was: 5.6 ± 1.3°, 4.6 ± 1.1°, 3.4 ± 1.2°, 2.3 ± 0.8°; The range of motion of the segment flexion, extension, lateral flexion and rotation were: 1.1 ± 0.2°, 1.6 ± 0.1°, 0.4 ± 0.2°, 0.2 ± 0.1° for L4–5 PLF model; The range of motion for posterior extension, lateral flexion, and rotation were: 1.0 ± 0.1°, 1.3 ± 0.1°, 0.3 ± 0.2°, 0.3 ± 0.8° for L4–5 PLIF model; Range of motion of flexion, extension, and lateral The range of motion for flexion and rotation for L4–5 MFF model was: 1.2 ± 0.3°, 1.1 ± 0.1°, 0.5 ± 0.2°, 0.3 ± 0.1°.

**Table 4:** L4–5 Postoperative Range of Movement(Unit:°)

Model	Flexion	Extension	Lateral Bending	Rotation
Intact Model	5.6 ± 1.3	4.6 ± 1.1	3.4 ± 1.2	2.3 ± 0.8
PLF Model	1.1 ± 0.2	1.6 ± 0.1	0.4 ± 0.2	0.2 ± 0.1
PLIF Model	1.0 ± 0.1	1.3 ± 0.1	0.3 ± 0.2	0.3 ± 0.8
Modified articular fusion model	1.2 ± 0.3	1.1 ± 0.1	0.5 ± 0.2	0.3 ± 0.1

**Fig. 5:**
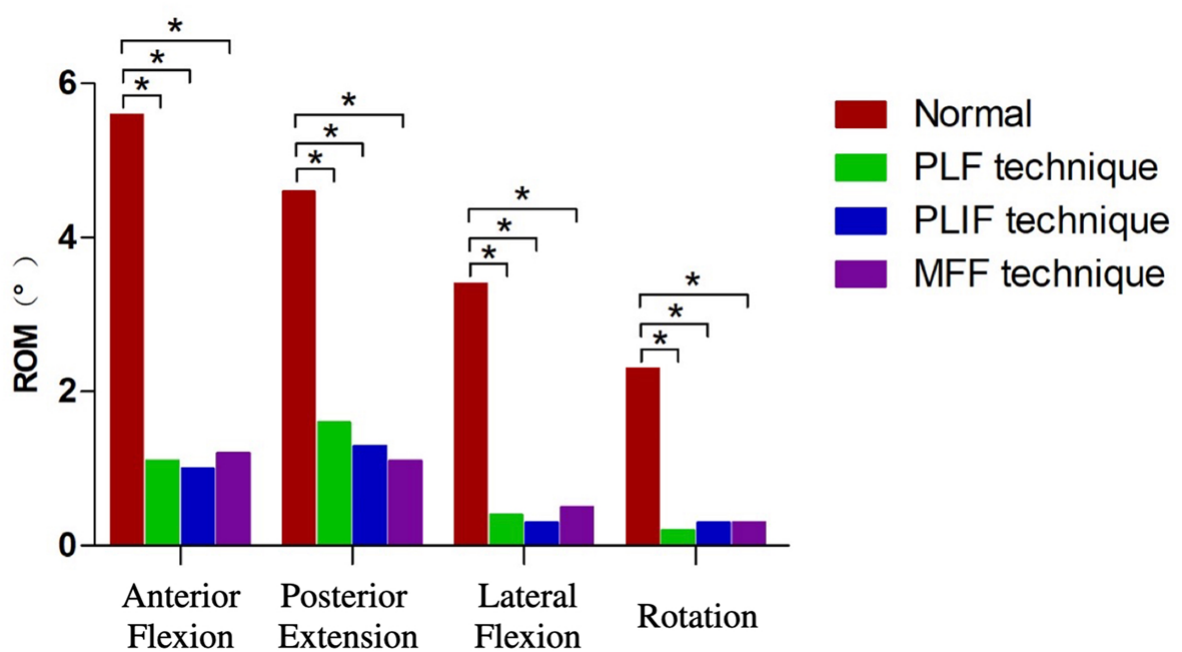
L4–5 Posterior Range of Motion. ^*^P < 0.05

As shown in Fig. 5, the range of motion of the three different fusion models under flexion, extension, left and right lateral flexion and rotation was significantly smaller than that of the normal intact lumbar model (*P* < 0.05), However, there was no significant difference in the range of activity between the PLF model, PLIF model and the MFF model (*P* > 0.05).

### Stiffness and stress distribution

**Fig. 6:**
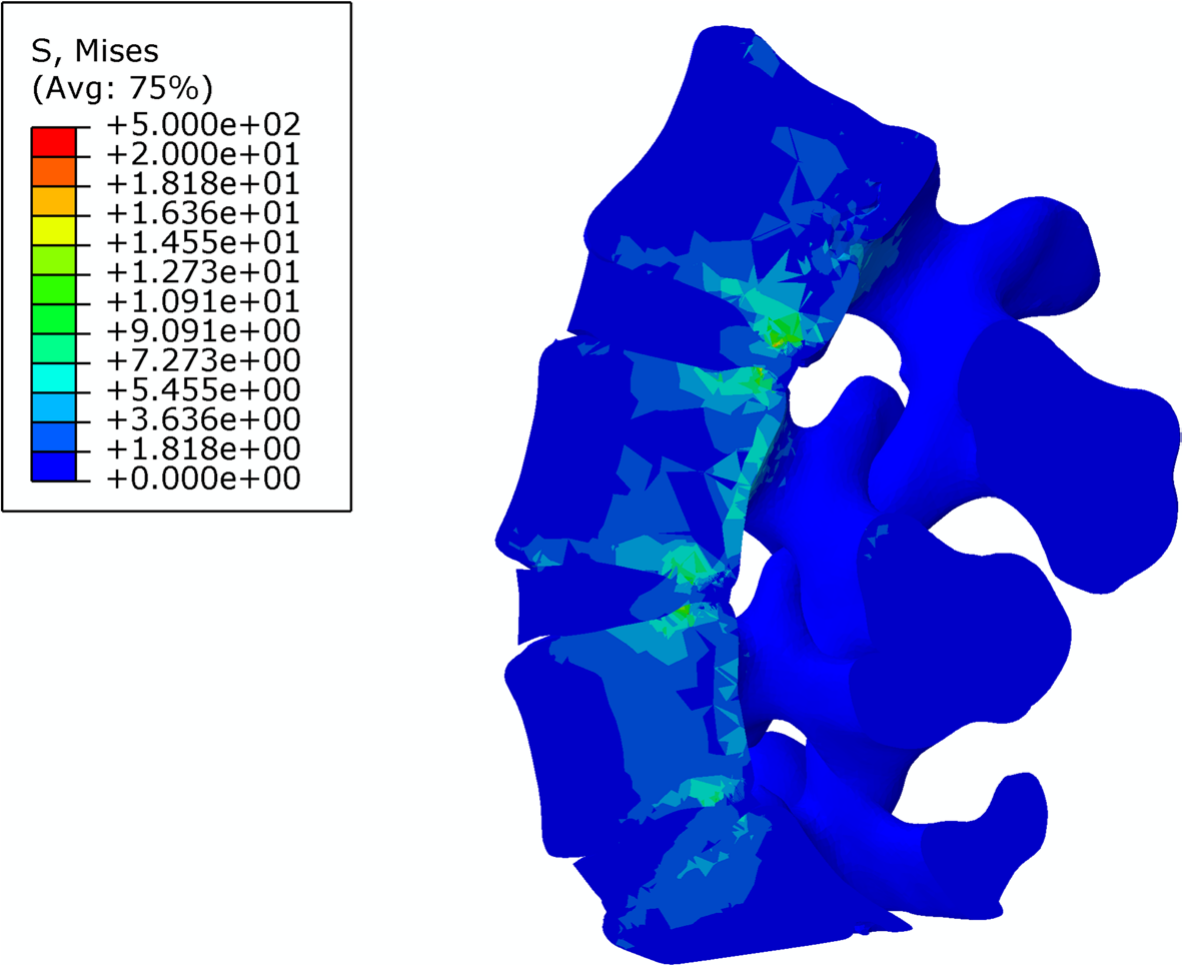
Lumbar Intact Model

**Fig. 7:**
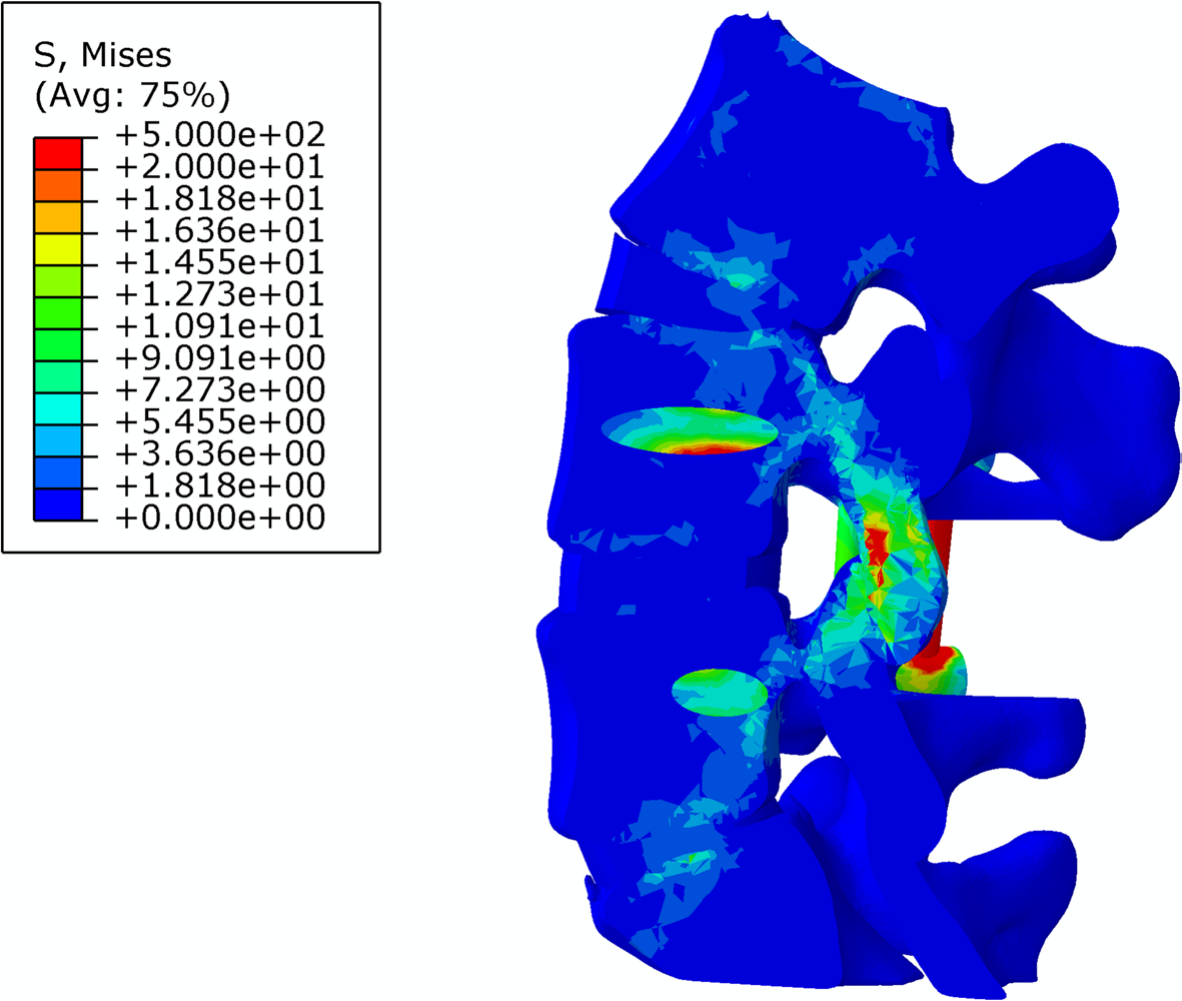
Stress distribution of PLF Model

**Fig. 8:**
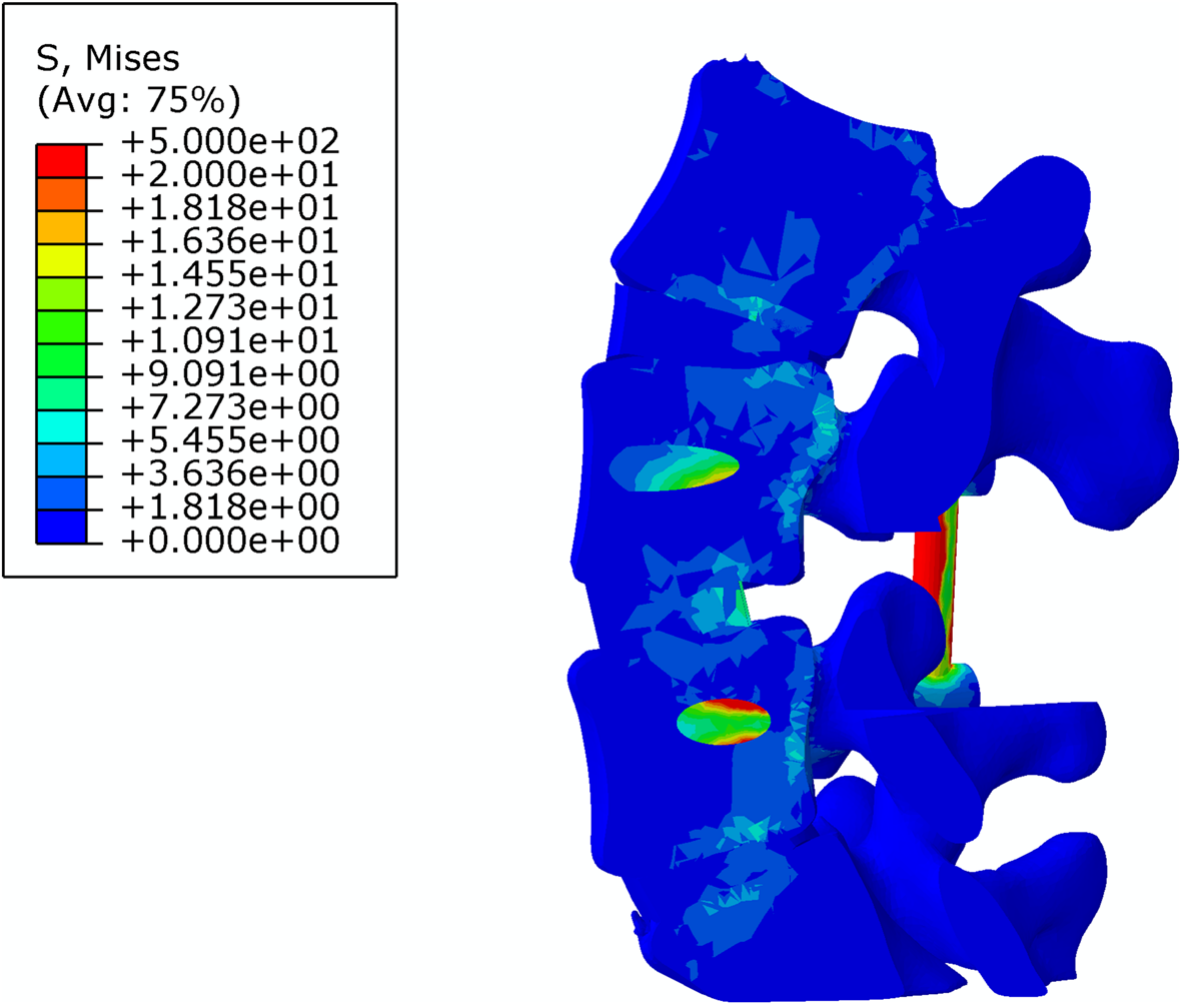
Stress distribution of PLIF Model

**Fig. 9:**
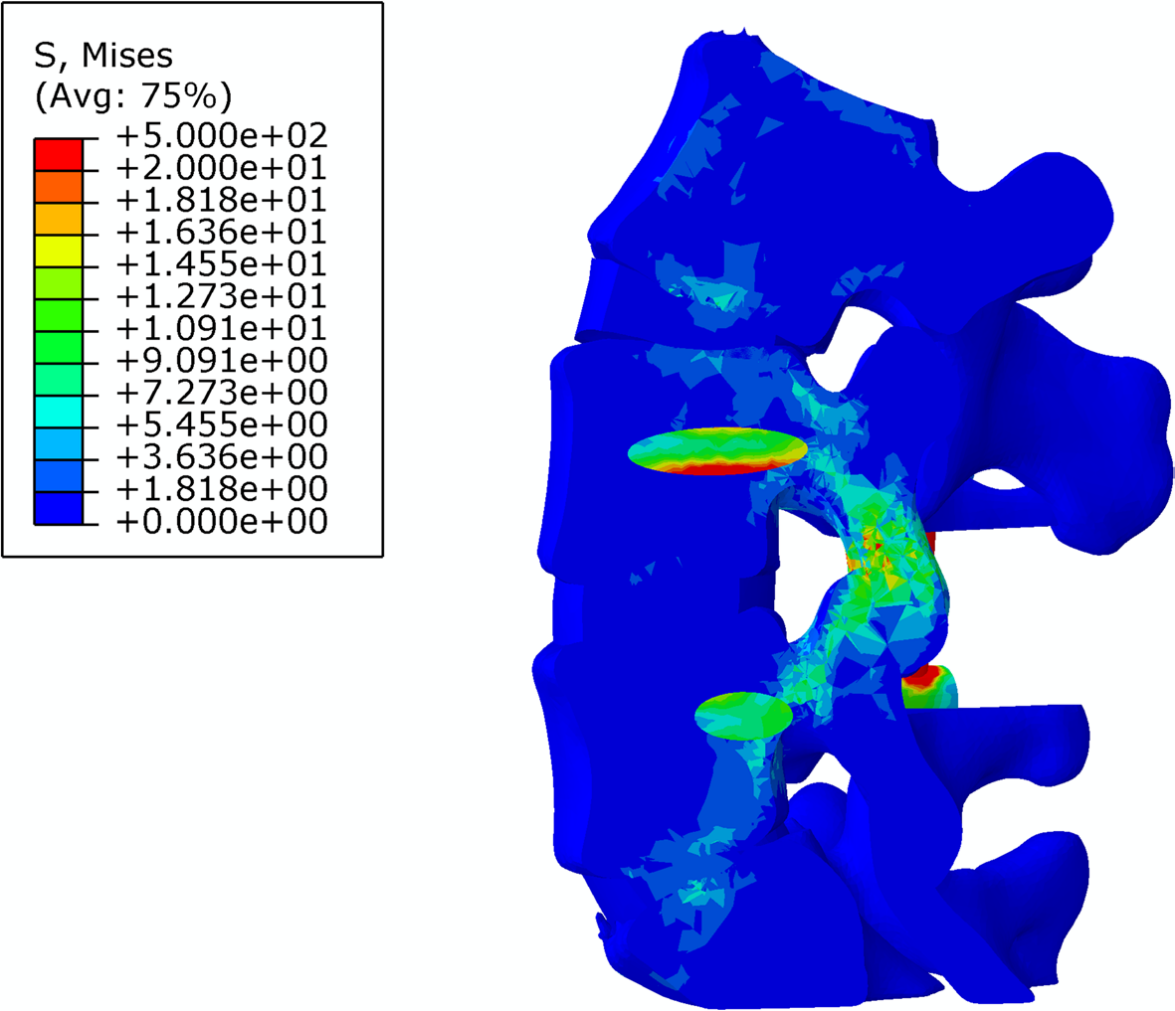
Stress distribution of MFF Model

The stiffness and stress distributions of the L4–5 segment of the intact model and the three different fusion post-operative models are shown in Table 5. The overall stiffness of the L4–5 segment of the intact model was 29.2 ± 2.1 N/mm (Fig. 6); the overall stiffness, the maximum stress at the fusion site and the maximum internal stress ratio of the L4–5 PLF were 56.9. ± 5.2 N/mm, 136.8 ± 4.5 MPa, 210.1 ± 8.3 MPa, respectively (Fig. 7); the overall stiffness, the maximum stress at the fusion site and the maximum internal stress ratio of the L4–5 segment PLIF model was 63.3 ± 5.5 N /mm, 180.7 ± 3.7 MPa, 83.3 ± 7.5 MPa (Fig. 8); the overall stiffness the maximum stress at the fusion site and the maximum internal stress ratio of the fixation of the L4–5 segment of MFF model were 53.1 ± 4.6 N/mm, 186.1 ± 3.1 MPa, 194.8 ± 10.2 MPa (Fig. 9).

**Table 5:** Stiffness and stress distribution of intact model and different postoperative fusion models

Model	Stiffness (N/mm)	Maximum Fusion Site Stress (MPa)	Maximum Internal Stress (MPa)
Intact Model	29.2 ± 2.1	–	–
PLF Model	56.9 ± 5.2	136.8 ± 4.5	210.1 ± 8.3
PLIF Model	63.3 ± 5.5	180.7 ± 3.7	83.3 ± 7.5
Modified articular fusion model	53.1 ± 4.6	186.1 ± 3.1	194.8 ± 10.2

**Fig. 10:**
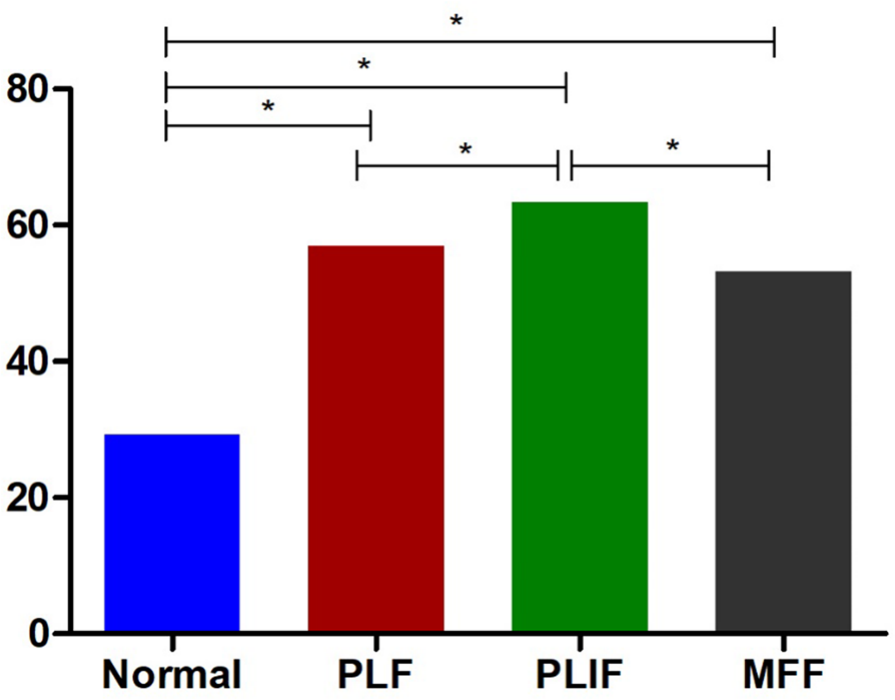
Stiffness of different models. ^*^P < 0.05

As shown in Fig. 10, the overall stiffness of the three different fusion models was significantly greater than the intact lumbar model (*P* < 0.05). The stiffness of the PLF and the MFF model were comparable (*P* > 0.05), but were smaller than the PLIF model (*P* < 0.05).

## Discussion

L4–5 segment is the common site for lumbar degenerative disease. Therefore, the L4–5 segment is used as the segment of the lesion to be analyzed. The finite element analysis technique is used to study the L4–5 segmental intact model and the postoperative model of different fusion techniques in order to investigate difference in postoperative stability between different fusion techniques. It was found that PLF technique, PLIF technique and MFF technique can achieve reliable stability of the fixed fusion segment and there is no significant difference in postoperative stability between the three fusion techniques.

Internal fixation fusion technology has become the basic technology for the treatment of lumbar disease. However, no matter what kind of internal fixation device is used, only short-term stability can be obtained. Only successful bone graft fusion can achieve long-term firmness and stability, thus maintaining the long-term effect after surgery. At present, the most widely used lumbar fusion techniques in clinical practice are: PLF and T/PLIF [22]. However, the current mainstream fusion technology either has a low fusion rate (such as PLF) [23], or is complicated to operate and has high surgical risk (such as T/PLIF) [24]. An ideal fusion technology in clinical practice that is both easy and reliable has yet to be found. In Comparison with PLF and T/PLIF, the fusion distance required for articular fusion is the smallest, so theoretically there is a high fusion rate. In addition, the facet joint is the posterior column structure of the spine in which the surgery does not involve operation of the anterior column. The technique does not need to pull the dural sac or nerve root, so the nerve damage can be minimized when the articular process fusion is performed. The results of our previous study indicate that the MFF technique is a fusion technique with simple operation, high fusion rate and good safety.

The academic community has always believed that the improvement of postoperative clinical symptoms and the fusion technology used in patients have limited association [23]. As long as the fusion is successful, different fusion techniques can achieve satisfactory surgical results. Moreover, there is no difference in the range of motion of the fixed fusion segment after surgery. Xu et al. [25] analyzed the biomechanical differences between different intervertebral fusion techniques and found no difference in the activity of fusion segments after PLIF and TLIF fusion. There is still no similar study to investigate the difference in the range of motion of the surgical segment after articular process fusion when compared with PLF and PLIF. The results of this study has shown that PLF, PLIF and MFF can all achieve reliable stability of the fixed fusion segment. The three fusion techniques have no significant difference in the stability of the surgical segment. Our previous clinical study has shown that MFF can not only significantly improve the clinical symptoms of patients but also have high fusion rate. It is safe, cost-effective, and postoperative stability in this study has proven to be no less than mainstream fusion technology such as PLF and PLIF.

The firm fusion can effectively reduce the stress of the internal fixation system, thereby reducing the incidence of internal fixation fatigue fracture, preventing internal fixation failure, and ensuring the long-term effect after surgery. The results of this study showed that in the PLF model, the maximum stress and internal fixation maximum stress ratio of the transverse fusion site was 136.8 ± 4.5 MPa, 210.1 ± 8.3 MPa; the maximum stress at the intervertebral fusion site and the maximum internal stress ratio in the PLIF model was 180.7 ± 3.7 MPa, 83.3 ± 7.5 MPa. The maximum stress and internal fixation maximum stress ratio of the articular process fusion site in the MFF model was 186.1 ± 3.1 MPa, 194.8 ± 10.2 MPa. All three fusion models establish an effective stress conduction path that disperses the stress of the internal fixation system. In PLIF model, most of stress is distributed to interbody cage, and stress on screw is relatively small. In PLF and MFF model, the internal fixation bear large part of loading stress, therefore, the internal fixation system of the PLIF model is significantly less powerful than the PLF and the MFF model. It is currently believed that the existing pedicle screw system is less likely to fail if the internal stress is less than 800Mpa [17], so the safety of the internal fixation system of the three fusion models is worthy of recognition.

The insufficiency of this study is it is far from enough to analyze and judge the problems encountered in the clinic only by the finite element method. Due to the shortcomings of the current finite element technology, the true degree of the simulated living body cannot be achieved. Clinical studies have to be further confirmed.

## Conclusion

In conclusion, MFF technique brings about reliable stability in the fixation fusion segment and shows no difference when compared with PLF technique and PLIF technique.

## Data Availability

The medical records and original image used during this study are available from the corresponding author on reasonable request.

## Abbreviations

MFF: Modified Facet joint Fusion
PLF: Posterior lateral Lumbar intertransverse Fusion
PLIF: Posterior Lumbar Interbody Fusion
ROM: Range of Motion

## References

[1] Friedenberg R (1969). "direct analysis" or "finite element analysis" in biology: a new computer approach. Curr Mod Biol.

[2] Balko B, Berger RL (1969). A direct finite element analysis method for particle mechanics: the three-body problem. Curr Mod Biol.

[3] Belytschko TB, Andriacchi TP, Schultz AB, Galante JO (1973). Analog studies of forces in the human spine: computational techniques. J Biomech.

[4] Hakim NS, King AI (1979). A three dimensional finite element dynamic response analysis of a vertebra with experimental verification. J Biomech.

[5] Más Y, Gracia L, Ibarz E, Gabarre S, Peña D, Herrera A. Finite element simulation and clinical follow-up of lumbar spine biomechanics with dynamic fixations. PLoS One. 2017;12(11):e0188328. 10.1371/journal.pone.0188328 PMC5706716 29186157

[6] Guo LX, Fan W (2018). Dynamic response of the lumbar spine to whole-body vibration under a compressive follower preload. Spine (Phila Pa 1976).

[7] Liu Q, Zhang J, Sun SC, Wang F (2017). Application of finite element method in spinal biomechanics. Zhongguo Gu Shang.

[8] Wang H, Zhao Y, Mo Z, Han J, Chen Y, Yu H, Wang Q, Liu J, Li C, Zhou Y (2017). Comparison of short-segment monoaxial and polyaxial pedicle screw fixation combined with intermediate screws in traumatic thoracolumbar fractures: a finite element study and clinical radiographic review. Clinics (Sao Paulo).

[9] Januszewski J, Beckman JM, Harris JE, Turner AW, Yen CP, Uribe JS (2017). Biomechanical study of rod stress after pedicle subtraction osteotomy versus anterior column reconstruction: a finite element study. Surg Neurol Int.

[10] Dijkerman ML, Overdevest GM, Moojen WA, Vleggeert-Lankamp CLA (2018). Decompression with or without concomitant fusion in lumbar stenosis due to degenerative spondylolisthesis: a systematic review. Eur Spine J.

[11] Liang HF, Liu SH, Chen ZX, Fei QM (2017). Decompression plus fusion versus decompression alone for degenerative lumbar spondylolisthesis: a systematic review and meta-analysis. Eur Spine J.

[12] Montgomery AS, Cunningham JE, Robertson PA (2015). The influence of no fault compensation on functional outcomes after lumbar spine fusion. Spine (Phila Pa 1976).

[13] Ren Z, Li Z, Li S, Xu D, Chen X (2020). Modified facet joint fusion for lumbar degenerative disease: case series of a fusion technique, clinical outcomes, and fusion rate in 491 patients. Oper Neurosurg (Hagerstown).

[14] de Kunder SL, van Kuijk SMJ, Rijkers K, Caelers I, van Hemert WLW, de Bie RA, van Santbrink H (2017). Transforaminal lumbar interbody fusion (TLIF) versus posterior lumbar interbody fusion (PLIF) in lumbar spondylolisthesis: a systematic review and meta-analysis. Spine J.

[15] Park MK, Park SA (2019). Clinical and radiological outcomes of unilateral biportal endoscopic lumbar interbody fusion (ULIF) compared with conventional posterior lumbar interbody fusion (PLIF): 1-year follow-up. Neurosurg Rev..

[16] Liu T, Khalaf K, Naserkhaki S, El-Rich M (2018). Load-sharing in the lumbosacral spine in neutral standing & flexed postures - a combined finite element and inverse static study. J Biomech.

[17] Fan W, Guo LX (2017). Influence of different frequencies of axial cyclic loading on time-domain vibration response of the lumbar spine: a finite element study. Comput Biol Med.

[18] Srinivas GR, Kumar MN, Deb A (2017). Adjacent disc stress following floating lumbar spine fusion: a finite element study. Asian Spine J.

[19] Choi HW, Kim YE (2017). Effect of lumbar fasciae on the stability of the lower lumbar spine. Comput Methods Biomech Biomed Engin.

[20] Zahari SN, Latif MJA (2017). The Effects of Physiological Biomechanical Loading on Intradiscal Pressure and Annulus Stress in Lumbar Spine: A Finite Element Analysis. J Healthc Eng..

[21] Panjabi MM, Oxland TR, Yamamoto I, Crisco JJ (1994). Mechanical behavior of the human lumbar and lumbosacral spine as shown by three-dimensional load-displacement curves. J Bone Joint Surg Am.

[22] Schnake KJ, Rappert D, Storzer B, Schreyer S, Hilber F, Mehren C (2019). Lumbar fusion-indications and techniques. Der Orthopade.

[23] Farrokhi MR, Rahmanian A, Masoudi MS (2012). Posterolateral versus posterior interbody fusion in isthmic spondylolisthesis. J Neurotrauma.

[24] Mimura T, Tsutsumimoto T, Yui M, Takahashi J, Kuraishi S, Misawa H (2021). Adjacent segment pathology following posterior lumbar interbody fusion for lumbar degenerative spondylolisthesis: a comparison between minimally invasive and conventional open approach. Spine J.

[25] Xu H, Tang H, Guan X, Jiang F, Xu N, Ju W, Zhu X, Zhang X, Zhang Q, Li M (2013). Biomechanical comparison of posterior lumbar interbody fusion and transforaminal lumbar interbody fusion by finite element analysis. Neurosurgery.

